# Expert Opinion on the Use of Probiotics in General Gynecological Conditions

**DOI:** 10.7759/cureus.80875

**Published:** 2025-03-20

**Authors:** Ameet Patki, Suchitra Pandit, Noushin Ashraf, Sanjay Makhwana, Bishwanath Ghosh Dastidar

**Affiliations:** 1 Obstetrics and Gynecology, Indian Society For Assisted Reproduction (ISAR), Mumbai, IND; 2 Obstetrics and Gynecology, Surya Hospitals, Mumbai, IND; 3 Obstetrics and Gynecology, Craft Hospital and Research Centre, Kochi, IND; 4 Obstetrics and Gynecology, Vasundhara IVF and Fertility Research Centre, Jodhpur, IND; 5 Obstetrics and Gynecology, Ghosh Dastidar (GD) Institute for Fertility Research, Kolkata, IND

**Keywords:** bacterial vaginosis, gsm, lactobacillus, oral probiotics, pcos, recurrent uti

## Abstract

Various gynecological conditions, including bacterial vaginosis, urinary tract infection, genitourinary syndrome of menopause, polycystic ovarian syndrome, and vulvovaginal candidiasis, impose a significant global burden, including among the Indian population. This expert opinion emphasizes the importance of oral probiotic supplementation in managing these conditions. A physical meeting with 14 experts was conducted on June 29-30, 2024, during which they highlighted that probiotics, particularly *Lactobacillus* species, have beneficial effects on restoring and maintaining healthy vaginal microbiota. Probiotics also promote vaginal health and aid in treating conditions such as bacterial vaginosis, vulvovaginal candidiasis, polycystic ovarian syndrome, and genitourinary syndrome of menopause.

Probiotics are proven effective in managing bacterial vaginosis by enhancing beneficial bacteria and reducing harmful ones. They help prevent and treat recurrent urinary tract infections by increasing lactobacilli levels, inhibiting *Candida* growth, and maintaining vaginal pH to prevent vulvovaginal candidiasis. Symptoms of genitourinary syndrome of menopause, such as vaginal dryness, itching, and recurrent urinary tract infections, can be alleviated through a combination of *Lactobacillus*-based probiotics and estrogen therapy. Experts also recommended probiotic supplementation for women with polycystic ovarian syndrome to improve both chemical and clinical pregnancy rates. Probiotics help modulate gut microbiota; improve blood glucose levels, insulin resistance, cholesterol, and androgen levels; restore the LH/FSH ratio; and enhance reproductive health. This expert opinion underscores the key role of *Lactobacillus* species in treating and preventing various gynecological disorders such as bacterial vaginosis, vulvovaginal candidiasis, genitourinary syndrome of menopause, urinary tract infection, and polycystic ovarian syndrome by restoring and maintaining vaginal microbiota, thus supporting overall feminine health.

## Introduction and background

Probiotics are live microorganisms that provide health benefits beyond basic nutrition when consumed in adequate amounts. The most common probiotic bacteria include *Lactobacillus*​​​​​* acidophilus, Lactobacillus casei, Lactobacillus reuteri, Lactobacillus*​​​​ *plantarum, L. casei GG, Bifidobacterium brevis, Bifidobacterium longum, Bifidobacterium infantis, Bifidobacterium animalis,* and *Streptococcus*​​ *thermophilus*. Some yeast strains, such as *Saccharomyces boulardii*, also exhibit probiotic properties [1]. Probiotics promote health through several mechanisms: enhancing the epithelial barrier of the gut to reduce intestinal permeability and prevent pathogen invasion; producing antimicrobial substances such as bacteriocins, organic acids, and hydrogen peroxide to inhibit the growth of harmful bacteria; and competing with pathogenic bacteria for adhesion sites on the intestinal mucosa to prevent their colonization. Additionally, probiotics modulate the host's immune response by stimulating the production of anti-inflammatory cytokines and suppressing pro-inflammatory cytokines, thus balancing the immune system and reducing inflammation. These actions contribute to maintaining a healthy gut microbiota and improving overall health [1-3].

Probiotics provide benefits such as modifying gut microbiota, alleviating nutritional intolerances like lactose intolerance, enhancing the bioavailability of certain macro- and micronutrients, and reducing allergic reactions in individuals with allergies [4]. They are effective in managing digestive health by modulating the gut microbiota, helping patients with conditions such as diarrhea, irritable bowel syndrome, and inflammatory bowel disease. In addition to aiding digestion, probiotics also strengthen the immune system by inhibiting infections through the production of natural antibodies and the enhancement of immune cell activity [3]. Moreover, the positive effects of probiotics on mental health are increasingly recognized, as gut health plays a central role in brain function. Probiotics may alleviate the symptoms of anxiety and depression in some individuals by addressing gut microbiome imbalance [5]. Mental health conditions are closely associated with an imbalance in gut microbiota, resulting in microbiome disruption (dysbiosis). The gut produces several neurotransmitters, including gamma-aminobutyric acid (GABA), serotonin, and glutamate, which are linked to anxiety and depressive symptoms. Given the established connection between mental health and gut microbiota, probiotics may help in managing these conditions. Probiotics are widely available as over-the-counter supplements, which lack strict regulation, to promote gut health, enhance mood, and help reduce stress [6]. Species-specific research has shown that probiotics can help lower cholesterol levels, reduce blood pressure, and support cardiovascular health. They can also assist in weight management and reduce obesity by influencing gut microbiota composition and metabolic processes [2,7].

This expert opinion emphasizes the potential of probiotics as a therapeutic option for various gynecological conditions, including bacterial vaginosis, urinary tract infection, genitourinary syndrome of menopause, polycystic ovarian syndrome, and vulvovaginal candidiasis. It highlights the significance of maintaining a balanced vaginal and endometrial microbiome, predominantly composed of beneficial *Lactobacillus* species. The consensus supports that probiotics can help restore microbial balance, reduce inflammation, and strengthen the genital epithelial barrier. This viewpoint is supported by increasing scientific evidence demonstrating the efficacy of probiotics in enhancing reproductive outcomes, offering a safe and noninvasive approach to managing microbial dysbiosis.

## Review

Methods

A physical expert meeting was held on June 29-30, 2024, to discuss the use of probiotics in women's reproductive health, focusing on general gynecological conditions. The panel consisted of 14 experts from across India, with diverse expertise in gynecology, obstetrics, and fertility. The primary objective was to explore the role of probiotics in enhancing women's health, including their potential benefits in gynecological treatments. Following extensive discussions, key insights were compiled into this document and shared with the experts for review and feedback. Based on their feedback and suggestions, this expert opinion report was finalized and circulated to all experts for approval.

Bacterial vaginosis

Prevalence and Impact

Bacterial vaginosis is a common condition among women of reproductive age, with varying prevalence rates globally. In India, studies show that bacterial vaginosis affects a significant portion of the population. For example, one study reported a prevalence of 44.8% among women of reproductive age [8]. Bhalla et al. reported a prevalence rate of 32.8%. In their study, the highest prevalence was observed in urban slum areas (38.6%), followed by rural areas (28.8%) and urban middle-class communities (25.4%) [9]. Under normal conditions, the vaginal environment is dominated by *Lactobacillus *bacteria, which maintain a healthy balance. However, bacterial vaginosis occurs when this balance is disrupted, leading to an overgrowth of anaerobic bacteria such as *Gardnerella vaginalis*, *Ureaplasma*, and *Mycoplasma*. This shift in the vaginal microbiota not only affects vaginal health but also increases local levels of pro-inflammatory cytokines, which damage the epithelial and mucosal barriers. If left untreated, even mild or asymptomatic bacterial vaginosis can escalate to more severe gynecological disorders, including endometritis, pelvic inflammatory disease, chronic vaginitis, and infertility [10].

Role of Probiotics

**Figure 1:**
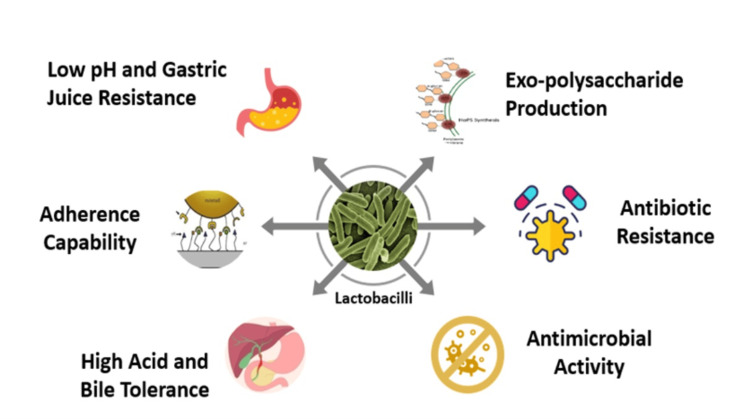
Lactobacilli as probiotics Image Credit: Authors' original creation.

Probiotics, particularly *Lactobacillus* strains, play a key role in restoring the natural vaginal flora (Figure 1). These beneficial bacteria produce lactic acid and other substances that inhibit the growth of pathogenic microbes. Clinical studies show that probiotics can improve treatment outcomes for bacterial vaginosis, reduce recurrence rates, and enhance overall vaginal health. This can be achieved through the use of probiotics alone or in combination with antibiotics [11].

Recommended Strains

Research highlights specific strains of *Lactobacillus *as particularly effective against bacterial vaginosis. In vitro studies have shown that these strains can inhibit the adhesion of *G. vaginalis* to the vaginal epithelium and produce hydrogen peroxide (H_2_O_2_), lactic acid, and bacteriocins, all of which suppress the growth of bacterial vaginosis-causing bacteria [12]. Clinical trials have demonstrated that intravaginal administration of *L. acidophilus* for 6-12 days, or oral administration of *L. acidophilus, Lactobacillus rhamnosus GR-1*, and *Lactobacillus fermentum* *RC-14* for two months, resulted in higher cure rates, reduced recurrence of bacterial vaginosis, and increased vaginal lactobacilli, restoring normal vaginal microbiota more effectively than placebo, acetic acid, or no treatment [13,14].

Clinical Studies

Several placebo-controlled, randomized trials have examined high doses (>10^7^ colony-forming units (CFUs)) of *Lactobacillus* strains, either singly or in combination, such as *L. rhamnosus, L. reuteri, L. salivarius, L.* *plantarum, L. acidophilus*, and *L. brevis*. Although individual studies were often underpowered to detect significant changes and variations in intervention lengths, an overall trend toward a cure for bacterial vaginosis was observed. These trials suggest that probiotic therapy is a promising and effective strategy for the management of bacterial vaginosis [15,16]. In a study by Reid et al., 64 healthy women took daily oral capsules of *L. rhamnosus GR-1*and *L.* *fermentum RC-14* for 60 days, with no adverse effects reported [13]. Treatment led to a significant improvement in vaginal microflora, with 37% of women achieving normal lactobacilli colonization compared to 13% on placebo (p = 0.02). Additionally, the treatment increased lactobacilli detection and significantly reduced yeast and coliforms at various time points. The study concluded that this probiotic combination is safe and effective in improving vaginal health [13]. A study by Mändar et al. assessed the efficacy of novel evidence-based probiotics in treating bacterial vaginosis and vulvovaginal candidiasis [17]. In this randomized, double-blind, placebo-controlled trial, 89 bacterial vaginosis and 93 vulvovaginal candidiasis patients, aged 18-50 years, were treated with either oral or vaginal probiotic capsules containing specific *Lactobacillus crispatus* strains or a placebo over three months. The results showed that both oral and vaginal probiotic capsules significantly alleviated bacterial vaginosis symptoms, with notable improvements in Nugent scores, discharge, and itching/irritation [17]. Another study by Reznichenko et al. investigated the effect of a mixture of three *Lactobacillus* species (containing *L. crispatus, L. brevis*, and *L. acidophilus*) on bacterial vaginosis recurrence in women treated with metronidazole [18]. In this phase 2 trial, women who received the probiotics had a significantly lower bacterial vaginosis recurrence rate (18.3%) compared to those on placebo (32.1%) (p = 0.014) and a longer period without bacterial vaginosis (p = 0.018). The study concluded that this probiotic combination effectively reduces bacterial vaginosis recurrence and prolongs remission [18].

Table 1 presents the expert consensus on the role of probiotics in treating bacterial vaginosis.

**Table 1:** Expert recommendations on probiotic use for managing bacterial vaginosis CFU: colony-forming unit.

Expert recommendations on the use of probiotics in treating bacterial vaginosis
Diagnosing bacterial vaginosis involves identifying a characteristic fishy odor, which is indicative of a microbial imbalance in the vaginal environment.
Probiotics, particularly *Lactobacillus *species, play a crucial role in restoring and maintaining natural vaginal microbiota balance.
Probiotics effectively support vaginal health; manage conditions like bacterial vaginosis, vulvovaginal candidiasis, and polycystic ovarian syndrome; and alleviate symptoms of genitourinary syndrome of menopause in postmenopausal women.
In bacterial vaginosis, probiotics increase beneficial bacteria, reduce harmful bacteria like *Gardnerella* and *Prevotella*, and stabilize the vaginal flora environment, supported by increasing evidence of their effectiveness in treatment.
The recommended strains for bacterial vaginosis include *L. acidophilus* *LA-14*, *L. crispatus*, *L. rhamnosus* *GR-1*, and *L. reuteri* *RC-14* for restoring the vaginal microbiota. The dosage should be 1-2 capsules daily, with each capsule containing approximately 10 billion CFUs for 4-6 weeks. A follow-up 1-2 months posttreatment is advised.
Probiotics should be considered a valuable treatment option for bacterial vaginosis.
Probiotics should be used along with antibiotics to improve treatment outcomes for bacterial vaginosis by reducing recurrence and enhancing cure/remission rates.

Urinary tract infections

Prevalence and Impact

Urinary tract infections are highly prevalent and have a significant impact, particularly among women. A study by Pardeshi et al. found a significantly higher prevalence of urinary tract infections in women (66.78%) compared to men (33.22%), which aligns with other research highlighting the higher frequency in women due to anatomical and physiological differences [19]. The use of antibiotics to treat urinary tract infections contributes to antibiotic resistance, both among uropathogenic microorganisms and other bacteria in the body. Antibiotic resistance in uropathogens is now a recognized issue, with repeated antibiotic treatments in women with recurrent urinary tract infections playing a major role in its development. This overuse also negatively affects the normal microbiota. As a result, antibiotic-free protective approaches have gained popularity worldwide, driven by the increasing resistance to antibiotics and growing patient preference for alternative treatments [20-22].

Role of Probiotics

Probiotics play a crucial role in preventing urinary tract infections, particularly in addressing recurrent infections in women, which are often caused by uropathogenic *Escherichia *​​​​​​*coli*. Evidence supports the use of probiotics as a strategy to combat these infections. Antibiotics used to treat urinary tract infections can reduce lactobacilli levels in the urinary system, potentially leading to antibiotic resistance and disrupting the natural barrier against infections [23].

Recommended Strains

Specific probiotic strains have been shown to be effective in preventing and treating recurrent urinary tract infections. *Lactobacillus* species, particularly *L. rhamnosus GR-1* and* L. reuteri*, have demonstrated promise in this regard [23]. A study by Falagas et al. reported that *L. rhamnosus GR-1* and *L. reuteri RC-14* (formerly known as *L. fermentum RC-14*) were the most effective among the lactobacilli strains for preventing urinary tract infections. Additionally, *L. casei *Shirota and *L. crispatus* *CTV-05* have shown efficacy in some studies. However, *L.* *rhamnosus *GG was found to be less effective in urinary tract infection prevention. Evidence from available studies suggests that probiotics are beneficial for preventing recurrent urinary tract infections in women and are considered safe [24].

Clinical Studies

Several key clinical studies demonstrate the efficacy of probiotics in preventing urinary tract infections. In a double-blind, placebo-controlled phase 2 study, Stapleton et al. investigated 100 premenopausal women who had experienced cystitis at least once in the previous 12 months [25]. The study divided patients into two groups: 48 received placebo treatment and 48 received intravaginal probiotics (LACTIN-V) containing *L. crispatus* (108 CFUs/mL) for 10 weeks following a cystitis episode. Results showed a significant decrease in recurrent urinary tract infections among patients who received the intravaginal *Lactobacillus *treatment compared to the placebo group [25]. A recent clinical trial by Gupta et al. investigated the use of prophylactic probiotics for preventing recurrent urinary tract infections [26]. In this double-blind study with 174 women, four groups were compared: placebo, oral probiotics, vaginal probiotics, and a combination of both. Results showed that the incidence of urinary tract infections was significantly lower in the vaginal probiotics (40.9%) and combination groups (31.8%) compared to the placebo (70.4%) and oral probiotics alone (61.3%). Additionally, these groups had fewer recurrences and a longer time to first urinary tract infection. Probiotic supplementation, especially with vaginal probiotics or in combination, proved effective and well-tolerated for preventing recurrent urinary tract infections [26]. A study by Mula et al. compared outcomes of antibiotic treatment with and without probiotics in 897 patients with lower urogenital tract infections [27]. Patients were divided into two groups: an intervention group receiving antibiotics with probiotics (*L. acidophilus* *LA3*, *B. animalis *ssp.* lactis* *BLC1*, and *L. casei* *BGP93*; 1 × 109 CFUs) (n = 460) and a comparison group receiving antibiotics alone (n = 437). Significant differences were observed in patient-reported improvement, with those receiving probiotics reporting better outcomes. The study concluded that adding probiotics to antibiotic therapy can reduce antibiotic side effects, enhance treatment efficacy, and help mitigate antibiotic resistance [27].

Table 2 presents the expert consensus on the role of probiotics in treating urinary tract infections.

**Table 2:** Expert recommendations on probiotic use for managing urinary tract infections

Expert recommendations on the use of probiotics in treating urinary tract infections
Recurrent urinary tract infections pose a significant burden on women's health, often caused by *E. coli*.
Probiotics help prevent and treat recurrent urinary tract infections by promoting healthy lactobacilli levels, and inhibiting *Candida* growth, thereby maintaining vaginal pH and preventing vulvovaginal candidiasis.
*Lactobacillus *species, such as *L. rhamnosus* *GR-1*, *L. crispatus*, and *L. reuteri*, are recommended for maintaining urinary tract health and preventing recurrent urinary tract infections. A typical regimen includes 1-2 capsules daily, typically around 10 billion CFUs per capsule, for 12 weeks. Follow-up is advised 3-6 months after completing the course to monitor for recurrence.
*Lactobacillus *species should be recommended to reduce the risk of antibiotic resistance and strengthen the natural defenses of the urinary system.
Prevention of recurrent urinary tract infections can be achieved through proper bladder emptying, judicious use of antibiotics, and incorporating probiotics into one's regimen.

Genitourinary syndrome of menopause

Prevalence and Impact

Genitourinary syndrome of menopause, previously referred to as vulvovaginal atrophy or atrophic vaginitis, is a condition resulting from the decline in ovarian function and subsequent decrease in estrogen levels during perimenopause. Over 50% of postmenopausal women experience a range of distressing symptoms such as vaginal dryness, pruritus, painful intercourse, urgency and frequency of urination, and urinary tract infections [28]. Research from India highlights that 80% of women report vaginal dryness, which is the most prevalent symptom of genitourinary syndrome of menopause [29].

Role of Probiotics

Recent research has highlighted the benefits of combining probiotics with estrogen to alleviate the symptoms associated with vulvovaginal atrophy. Probiotics have shown promise in managing genitourinary syndrome of menopause symptoms effectively [28].

Recommended Strains

Specific probiotic strains have been proven to be effective in treating genitourinary syndrome of menopause. In a randomized controlled study, oral probiotics *L. rhamnosus GR-1* and *L. reuteri RC-14* were found to significantly reduce the Nugent score and improve genitourinary syndrome of menopause symptoms in postmenopausal women [30].

Clinical Studies

Several key clinical studies support the efficacy of probiotics in managing genitourinary syndrome of menopause symptoms. One notable study by Petricevic and colleagues demonstrated that the use of *L. rhamnosus GR-1* and *L. reuteri RC-14* led to significant improvements in genitourinary syndrome of menopause symptoms (p = 0.0001) [30]. A recent study by Vicariotto et al. investigated the benefits of probiotics on vaginal well-being and microbiota in postmenopausal women. This prospective trial involved 50 healthy women aged 45-65, who took a daily supplement containing *Lactiplantibacillus plantarum* *PBS067*, *B. animalis *ssp.* lactis BL050*, and *Lacticaseibacillus rhamnosus LRH020* for 28 days. Results showed a 50% improvement in the Vaginal Health Index score and significant reductions in inflammatory cytokines: IL-6 (87.8%), IL-1β (57.6%), and TNF-α (40.8%). Additionally, the probiotic intervention restored vaginal microbiota, increasing lactobacilli abundance. These findings suggest that this probiotic combination is effective in improving vaginal health in postmenopausal women [31].

Table 3 presents the expert consensus on the role of probiotics in treating genitourinary syndrome of menopause.

**Table 3:** Expert recommendations on probiotic use for managing genitourinary syndrome of menopause CFU: colony-forming unit.

Expert recommendations on the use of probiotics in treating genitourinary syndrome of menopause
Combining probiotics with estrogen can alleviate symptoms of genitourinary syndrome of menopause like vaginal dryness, pruritis, and recurrent urinary tract infection.
Prevention of recurrent urinary tract infection can be achieved by emptying of full bladder when needed, discrete use of antibiotics, and addition of probiotics.
*L. rhamnosus* *GR-1*, *L. fermentum,* and *L. reuteri* *RC-14* are recommended for the treatment of genitourinary syndrome of menopause, with a daily dosage of 1-2 capsules, typically around 10 billion CFUs per capsule, for eight weeks. A follow-up 1-2 months after completing the course is advised to monitor for vaginal health.

Vulvovaginal candidiasis

Prevalence and Impact

Vaginal candidiasis, commonly referred to as vulvovaginal candidiasis, is a prevalent fungal infection affecting around 75% of women at least once in their lifetime. In India, research has revealed that the prevalence of laboratory-confirmed vulvovaginal candidiasis among women of reproductive age ranges from 10% to 35% [32]. It is characterized by symptoms such as leukorrhea (a white discharge), severe itching, vulvar redness, painful urination, and discomfort during intercourse. This condition is typically managed with antifungal treatments [33,34].

Role of Probiotics

Probiotics, particularly *Lactobacillus *species, play a crucial role in both the prevention and treatment of vulvovaginal candidiasis. They help maintain a healthy vaginal environment by producing lactic and acetic acids, and H_2_O_2_. These substances help keep the vaginal pH around 4.5, inhibiting the growth of *Candida albicans* and other pathogenic organisms. This protective effect is enhanced by the competition for nutrients and mucosal receptors, as well as the production of bacteriocins by probiotics [35].

Recommended Strains

Probiotics, particularly certain strains of *Lactobacillus*, play a crucial role in maintaining vaginal pH at levels that inhibit the growth of *Candida *species, thus helping prevent vulvovaginal candidiasis. Lactobacilli such as *L.* *acidophilus *and *L. rhamnosus* produce lactic acid, which lowers vaginal pH. This acidic environment inhibits the overgrowth of *C. albicans* and other pathogenic organisms that thrive in a more alkaline setting [36,37]. By maintaining this balanced pH, probiotics help prevent the recurrence of vulvovaginal candidiasis and contribute to overall vaginal health [35]. Specific strains of *Lactobacillus*, such as *L. gasseri *and *L. crispatus*, have been shown to be particularly effective in inhibiting *C. albicans*. These strains help restore and maintain the normal vaginal flora, thereby reducing the incidence and recurrence of vulvovaginal candidiasis [38].

Clinical Studies

Several key studies have demonstrated the efficacy of probiotics in managing vulvovaginal candidiasis. For instance, research has shown that probiotics are significantly more effective than placebos in treating vulvovaginal candidiasis. *Lactobacillus *strains, by producing lactic acid, acetic acid, and hydrogen peroxide, not only maintain a low pH but also create an environment hostile to *Candida* albicans and other pathogens [38]. A study by Davar et al. investigated the effect of probiotics on the recurrence of vulvovaginal candidiasis after an initial treatment with oral fluconazole [39]. In this randomized, double-blind trial, 59 vulvovaginal candidiasis patients were treated with a single dose of 150-mg fluconazole and then divided into two groups: one received probiotics and the other a placebo. Over six months, recurrence rates were significantly lower in the probiotic group (7.2%) compared to the placebo group (35.5%). Statistical analysis showed a significant difference (p = 0.01), with an odds ratio of 0.14, indicating that probiotics significantly reduced vulvovaginal candidiasis recurrence. The study suggested that adding probiotics to antifungal treatment can be highly effective in lowering recurrence rates [39].

Table 4 presents the expert consensus on the role of probiotics in treating vulvovaginal candidiasis.

**Table 4:** Expert recommendations on probiotic use for managing vulvovaginal candidiasis

Expert recommendations on the use of probiotics in treating vulvovaginal candidiasis
Probiotics prevent and treat recurrent urinary tract infections by promoting healthy lactobacilli levels and inhibiting *Candida* growth, thereby maintaining vaginal pH and preventing vulvovaginal candidiasis.
The recommended strains are *L. rhamnosus GR-1* and *L. reuteri RC-14*, with a daily dosage of 1-2 capsules, each containing approximately 10 billion CFUs. The treatment should last for 4-6 weeks. A follow-up 1-2 months posttreatment is advised.
When managing vulvovaginal candidiasis, maintaining proper genital hygiene is crucial before initiating probiotic therapy. Good hygiene practices help reduce the risk of infection and ensure the effectiveness of probiotics in restoring a healthy vaginal microbiota.

Polycystic ovarian syndrome

Prevalence and Impact

Polycystic ovarian syndrome is marked by excessive androgen production from the ovaries, resulting in symptoms such as chronic anovulation, ovarian cysts, acne, hirsutism, insulin resistance, and weight gain. It is a prevalent cause of infertility among women. According to the World Health Organization, polycystic ovarian syndrome is estimated to impact between 8% and 13% of women in their reproductive years [40]. The prevalence of polycystic ovarian syndrome in India varies between 3.7% and 22.5%, depending on the specific population examined and the diagnostic criteria applied [41]. The exact causes of polycystic ovarian syndrome remain unclear, as it is a complex, multigenetic disorder [34].

Role of Probiotics

The effectiveness of probiotics and synbiotics in managing polycystic ovarian syndrome is well-supported by research. Studies indicate that supplementation with probiotics or synbiotics can improve hormonal imbalances, reduce inflammation, and address lipid metabolism issues associated with polycystic ovarian syndrome. Additionally, research shows that these supplements may help manage weight, lower BMI, improve insulin levels, and reduce HOMA-IR, potentially playing a role in protecting fertility [42,43]. Probiotics have been shown to alleviate polycystic ovarian syndrome symptoms by modulating gut microbiota. They increase levels of beneficial bacteria such as *Bifidobacterium *and *Lactobacillus*, thereby restoring microbiota balance [44].

Recommended Strains

Specific strains of probiotics have been effective in managing polycystic ovarian syndrome symptoms. They work by adjusting the gut microbiota, boosting levels of beneficial bacteria such as *Bifidobacterium *and *Lactobacillus*, restoring a healthy balance of gut microbes, decreasing intestinal permeability, and reducing the transfer of harmful lipopolysaccharides from the gut into the bloodstream [45].

Clinical Studies

A recent meta-analysis of 17 randomized controlled trials involving 1,049 participants demonstrated that probiotic supplementation significantly lowered fasting blood glucose, insulin levels, and insulin resistance in women with polycystic ovarian syndrome. Additionally, it was associated with decreased levels of total cholesterol, low-density lipoprotein (LDL) cholesterol, and triglycerides, although high-density lipoprotein (HDL) cholesterol levels remained unchanged. Hormonal improvements were also noted, with significant reductions in luteinizing hormone (LH) and testosterone levels [44]. Furthermore, the first randomized controlled trial examining sexual function in polycystic ovarian syndrome patients found that an eight-week probiotic regimen improved the LH/follicle-stimulating hormone (FSH) ratio, chemical and clinical pregnancy rates, sexual function, and body satisfaction [46]. A study by Ghanei et al. assessed the impact of probiotic supplementation on clinical and immunological parameters in patients with polycystic ovarian syndrome through a randomized controlled trial [47]. The study compared a probiotic group, which received four strains of *Lactobacillus*, with the placebo group receiving maltodextrin. Key immunological markers (IL-6, IL-10, TNF-α, and hs-CRP) were measured before and after the trial. Probiotic supplementation significantly increased IL-10 levels compared to the placebo. Both groups experienced a notable decrease in hs-CRP and IL-6 levels, but TNF-α levels remained unchanged. These findings suggested that *Lactobacillus* supplementation can modulate inflammation in polycystic ovarian syndrome patients [47].

Zhang et al. conducted a meta-analysis of randomized controlled trials to evaluate the effects of probiotic supplementation on glucose homeostasis in polycystic ovarian syndrome patients [48]. Out of 825 reports, 11 trials were included. The pooled data showed that probiotics significantly reduced fasting blood glucose (SMD = −0.40), insulin levels (SMD = −0.57), and insulin resistance (SMD = −0.64) while increasing the quantitative insulin sensitivity check index (QUICKI) (SMD = 0.58). Notably, the impact on fasting blood glucose diminished with higher baseline BMI and mean age, and a greater number of bacterial species and higher doses were more effective in improving QUICKI. The study suggested that probiotics can aid in managing glucose homeostasis in polycystic ovarian syndrome patients [48]. Azizi-Kutenaee et al. conducted a double-blind trial to evaluate the effects of oral probiotics on sexual function in polycystic ovarian syndrome patients treated with letrozole [46]. Women were assigned to receive Lactofem with letrozole or letrozole alone, with both groups also taking folic acid. After two months, the Lactofem group showed significant improvements in sexual function, with higher pregnancy rates (10% vs. 0%, p = 0.05) and better body image scores (p < 0.01) compared to the letrozole-only group. The study suggested that adding probiotics can enhance sexual function and body image in polycystic ovarian syndrome patients on letrozole [46].

Table 5 presents the expert consensus on the role of probiotics in treating polycystic ovarian syndrome.

**Table 5:** Expert recommendations on probiotic use for managing polycystic ovarian syndrome CFU: colony-forming unit.

Expert recommendations on the use of probiotics in treating polycystic ovarian syndrome
Probiotics should be considered a potential therapeutic approach for managing polycystic ovarian syndrome.
Probiotic supplementation should be recommended for women with polycystic ovarian syndrome to improve chemical and clinical pregnancy rates.
In polycystic ovarian syndrome, probiotics modulate gut microbiota; improve blood glucose, insulin resistance, cholesterol, and androgen levels; restore LH/FSH ratio; and improve overall reproductive health.
The recommended strains are *L. crispatus*, *L. acidophilus*, *B. lactis* *BB-12*, and *L. reuteri*, with a daily dose of 1-2 capsules, containing approximately 10-20 billion CFUs. The advised treatment duration is 12 weeks. A follow-up three months posttreatment is advised to assess hormonal balance and symptom improvement.
In the context of polycystic ovarian syndrome, incorporating lifestyle changes, such as a balanced diet, regular physical activity, weight management, and stress reduction, is vital alongside other treatments to manage symptoms and improve overall health outcomes.

Safety and efficacy

Safety Profile

Probiotics are generally considered safe for use in gynecological conditions. They are often used to support vaginal health by maintaining a balanced microbiota and preventing infections. Studies indicate that probiotics have a low risk of adverse effects, with most individuals experiencing no significant issues [49,50].

Potential Side Effects

While rare, some individuals may experience mild side effects such as flatulence, bloating, or digestive discomfort when starting probiotics. There are few contraindications, but those with compromised immune systems or severe underlying health conditions should consult a healthcare provider before use [51].

Long-Term Use

Long-term use of probiotics is generally safe for most people. However, concerns have been raised about potential impacts on antibiotic resistance and the balance of gut microbiota over extended periods [52]. Regular monitoring and consultation with a healthcare professional are recommended for prolonged use to ensure continued safety and efficacy.

## Conclusions

*Lactobacillus *species, especially among probiotics, play an important role in the treatment and prevention of various gynecological disorders. They help restore and maintain the natural vaginal microbiota balance, supporting vaginal health and managing conditions such as bacterial vaginosis, vulvovaginal candidiasis, and polycystic ovarian syndrome. Evidence suggests that probiotics may alleviate symptoms of genitourinary syndrome of menopause in postmenopausal women and promote urinary tract health, preventing recurrent urinary tract infections. While clinical recommendations are limited by available evidence, probiotic interventions appear to be an effective alternative or adjunctive treatment option for urogenital infections. Probiotics should be considered advantageous in bacterial vaginosis treatment, particularly when administered alongside antibiotics, to enhance treatment efficacy and prevent recurrence. *Lactobacillus *strains* such as L. rhamnosus GR-1* and *L. reuteri* have been recommended for urinary tract infection prevention and maintaining urinary tract health, also reducing the risk of antibiotic resistance. Probiotic supplementation has also been shown to improve pregnancy rates in women with polycystic ovarian syndrome. Overall, probiotics represent a promising treatment option for a range of gynecological conditions.
